# Balancing drone adoption with infrastructure readiness in China

**DOI:** 10.1016/j.xinn.2026.101439

**Published:** 2026-05-27

**Authors:** Shixiong Zhou, Dabin Xue, Shuai Jia, Xiqun (Michael) Chen

**Affiliations:** 1 Thrust of Intelligent Transportation, The Hong Kong University of Science and Technology (Guangzhou), Guangzhou 511453, China; 2 School of Vehicle and Mobility, Tsinghua University, Beijing 100084, China; 3 State Key Laboratory of Solar Activity and Space Weather, National Space Science Center, Chinese Academy of Sciences, Beijing 100190, China; 4 Department of Civil and Environmental Engineering, The Hong Kong University of Science and Technology, Hong Kong, China; 5 Institute of Intelligent Transportation Systems, College of Civil Engineering and Architecture, Zhejiang University, Hangzhou 310058, China

## Main text

**Figure 1:**
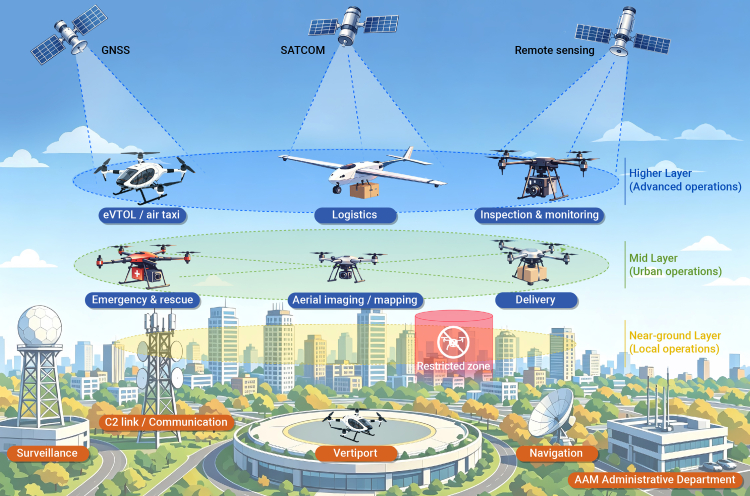
Low-altitude economy ecosystem Unmanned aircraft, satellites, and ground control systems operate within dense urban airspace, requiring integrated coordination of communication, navigation, surveillance, and advanced air mobility (AAM) administrative departments for safe and efficient operations.

The low-altitude economy is reshaping the future of aviation. Covering activities below 1,000 m, and extendable to 3,000 m, it integrates platforms from delivery drones to electric air taxis, marking a fundamental transformation in airspace use. In 2025, the total number of registered drones surpassed 3.28 million, with cumulative flight hours reaching 45.3 million, marking a nearly 70% year-on-year increase.^1^ The low-altitude economy ecosystem, as illustrated in Figure 1, encompasses an intricate network of unmanned aircraft, satellite-based communications and navigation, ground infrastructure, and urban airspace, all of which demand seamless coordination. To ensure safety and enable sustainable growth, China’s commitment is clear. In April 2024, the low-altitude economy was first mentioned in the government work report, a milestone widely recognized as “year one” of systematic development. In 2025, the “Government Work Report” emphasized promoting “safe and healthy development” of the low-altitude economy and conducting large-scale application demonstrations. In March 2026, the low-altitude economy was included in the Government Work Report for the third consecutive year, and its positioning was further upgraded to be among the four “emerging pillar industries” along with integrated circuits, aerospace, and biomedicine.

To fully realize this potential, several developmental challenges require systematic attention. The rapid expansion of low-altitude operations presents new management challenges that require evolutionary approaches to airspace systems designed for traditional civil aviation. Despite this, unmanned aerial vehicles (UAVs) continue to operate under regulations crafted for an era when low-altitude flights were rare and uniform, highlighting areas where regulatory frameworks require modernization to accommodate emerging technologies.

## Managerial challenge

Traditional airspace management is designed for high-altitude operations above 6,000 m and for controlled terminal zones around airports, relatively structured environments characterized by standardized aircraft types, mature technologies, and well-defined separation rules. In contrast, low-altitude operations introduce a far more complex and dynamic environment. UAVs range from 250-g consumer drones to 2.4-ton passenger electric vertical take-off and landing (eVTOL) aircraft, flying through dense urban corridors, mountainous regions, and coastal areas, each presenting unique challenges in terrain, weather, and electromagnetic interference.^2^ Their operational objectives are diverse. For example, high-speed mission demands differ fundamentally from low-speed infrastructure inspections. As a result, traditional weight-based and mode-based separation rules, effective for conventional aviation, are no longer adequate for this heterogeneous and fast-evolving environment.

## Coordination mechanisms

Low-altitude operations span multiple jurisdictions and require coordination among various government departments, from airworthiness certification and safety oversight to operational licensing and emergency response protocols. This multi-stakeholder environment represents a common challenge faced by aviation authorities worldwide as they adapt to emerging low-altitude activities. The National Airspace Management Committee serves as a coordination platform, facilitating dialogue between civil aviation authorities, military airspace management, and local government agencies. Crucially, to move beyond static administrative boundaries, this platform must facilitate dynamic airspace configuration, allowing algorithms to securely and swiftly reallocate airspace blocks based on real-time demand. As low-altitude activities expand from rural cargo operations to urban passenger transport, coordination mechanisms must accommodate varying risk profiles while maintaining system-wide coherence and efficiency.

## Regulatory framework deployment

Like many countries worldwide, China is developing a comprehensive legal framework for low-altitude airspace regulation as this sector rapidly evolves. The existing Civil Aviation Law provides only limited coverage of such operations, while the Aviation Law remains in draft form. In practice, regulation depends on a patchwork of administrative documents, such as the Basic Rules of Flight, Regulations on General Aviation Flight Control, and Interim Regulations on Unmanned Aircraft System Management, which often overlap or even contradict one another. Discrepancies also emerge between national and provincial regulations. For example, Hunan Province’s General Aviation Regulations authorize provincial compilation of aeronautical charts, directly conflicting with national rules that assign this responsibility to the Civil Aviation Information Center. This fragmentation stands in stark contrast to international frameworks. ICAO (International Civil Aviation Organization) Annex 2 provides unified rules of the air globally, and EASA (European Union Aviation Safety Agency) regulations establish harmonized standards across member states.

## Pathway forward

Effective reform must treat low-altitude airspace management as an integrated system, encompassing regulation, infrastructure, and operations, rather than merely an extension of economic development. It should operate independently while aligning with national strategies for technological innovation and industrial development, ensuring safety and energy efficiency^3^ as well as addressing critical social acceptance factors such as noise pollution, visual impact, and privacy in dense urban corridors. From this perspective, several key priorities are as follows.

### Unified legislation

Aviation laws should be accelerated to establish clear legal frameworks for low-altitude operations, resolving current regulatory fragmentation and conflicts. This process requires a refined airspace classification system that designates specific volumes for unmanned aircraft system traffic management (UTM) or U-space operations. International experience demonstrates the value of comprehensive legislation. For example, FAA (Federal Aviation Administration) Part 107 regulations provide a cohesive framework for unmanned aircraft systems, and EASA’s EU Regulation 2019/947 establishes proportionate, risk-based rules for drone operations.

### Infrastructure optimization

Adaptive management systems for low-altitude infrastructure should be developed that accommodate demand fluctuations, aircraft heterogeneity, and dynamic airspace transitions across diverse scenarios, including cargo delivery, emergency response, and inspection operations. Critical infrastructure components, such as vertiports, charging stations, and communication networks, require operational frameworks distinct from traditional aviation facilities. Crucially, these physical components must be integrated with robust digital infrastructure, utilizing communication, navigation, and surveillance (CNS) systems to overcome urban signal obstructions, especially during severe space weather events.^4^

### Clarified authority

Define distinct roles for military, civil aviation, and local authorities, with a strengthened coordination mechanism that can make binding decisions, not merely facilitate discussion. The model should draw on international best practices while respecting China’s specific civil-military integration requirements.

### Technology-enabled management

There should be investment in digital systems centered on UTM frameworks. By shifting from traditional human-centric control to a machine-centric paradigm, these systems can utilize highly automated, algorithmic flight scheduling and dynamic capacity evaluation. Furthermore, incorporating robust optimization strategies is essential to mitigate operational uncertainties like micro-weather changes and urban wind turbulence across highly heterogeneous fleets. Advanced urban air mobility concepts require sophisticated traffic management systems capable of real-time conflict detection and resolution, underpinned by reliable conflict detection and resolution (CDR) systems to safely manage the extreme heterogeneity of low-altitude traffic.

### Phased standards development

Performance-based regulations should be created that accommodate diverse aircraft types and missions while maintaining safety rather than applying inappropriate commercial aviation rules. Adapting globally recognized methodologies, such as the specific operations risk assessment (SORA), to China’s unique geographic and regulatory environment will be instrumental here. Safety standards must evolve alongside technology, particularly for emerging battery technologies that present unique certification challenges.^5^

The low-altitude economy represents more than technological innovation. Instead, it embodies a fundamental rethinking of airspace management in an era of ubiquitous aviation. China’s experience offers valuable lessons for nations worldwide as drones and air taxis become commonplace. China’s systematic approach to low-altitude economy development offers valuable insights for global aviation evolution. As technologies advance, adaptive governance frameworks will continue to evolve alongside industry innovation.

## Funding and acknowledgment

This work is supported by the 10.13039/501100001809National Natural Science Foundation of China (grants 72525009, 72431009, and 72542013), the Strategic Public Policy Research Funding (SPPRF) Scheme of the Government of the Hong Kong SAR (S2024.A7.022.24S), and the Specialized Research Fund for State Key Laboratory of Solar Activity and Space Weather. The authors would like to clarify that any information, opinions, findings, conclusions, or recommendations in this paper do not represent the views of the Government of the Hong Kong SAR and/or the SPPRF Project Assessment Panel.

## Declaration of interests

The authors declare no competing interests.
